# Comparative Evaluation of Crushed Almonds, Peanut Skins, and Water Hyacinth as Alternative Feed Resources for Ruminants: Fermentation Responses and Methane Mitigation Potential

**DOI:** 10.3390/ani16030489

**Published:** 2026-02-04

**Authors:** Ahmed O. Matti-Alapafuja, Eslam Ahmed, Ana Maria da Costa Goncalves Noronha, Rukayat O. Matti-Sanni, Masaaki Hanada, Naoki Fukuma, Takehiro Nishida

**Affiliations:** 1 Graduate School of Animal Husbandry, Obihiro University of Agriculture and Veterinary Medicine, Inada, Obihiro 080-8555, Japan; profahmed01@gmail.com (A.O.M.-A.); agoncalvesnoronha@gmail.com (A.M.d.C.G.N.); 2 Department of Animal Behavior and Management, Faculty of Veterinary Medicine, Qena University, Qena 83523, Egypt; eslam_kh@vet.svu.edu.eg; 3 Department of Botany, University of Lagos, Lagos 23401, Nigeria; rukayatsannimatti@gmail.com; 4 Department of Life and Food Science, Obihiro University of Agriculture and Veterinary Medicine, Inada, Obihiro 080-8555, Japan; hanada@obihiro.ac.jp (M.H.); n.fukumax@obihiro.ac.jp (N.F.); 5 Research Center for Global Agromedicine, Obihiro University of Agriculture and Veterinary Medicine, Inada, Obihiro 080-8555, Japan

**Keywords:** rumen fermentation, methane mitigation, alternative feed resources, agro-industrial by-products, in vitro digestibility, sustainable livestock production

## Abstract

Agro-residues and aquatic biomass are potential sustainable ruminant feeds. However, their effects on rumen digestibility, fermentation, and greenhouse gas emissions remain insufficiently characterized. This study used two in vitro batch culture experiments to evaluate crushed almonds, peanut skins, and water hyacinth as feed additives (1–10%) and grass hay replacements (25–100%). Their chemical composition varied significantly. Crushed almonds are rich in crude protein (22.47%) and ether extract (38.38%). Peanut skins contained moderate protein (13.87%) and high fiber and lignin, while water hyacinth had the lowest protein (7.27%) and highest fiber and ash fractions. All ingredients had no effect on fermentation or methane production when used as additives, whereas grass hay replacement induced marked ingredient-specific responses. Crushed almonds showed the highest in vitro dry matter digestibility (IVDMD), increased propionate, and lowered the A:P ratio; however, methane production increased by 12%. Peanut skins sharply reduced IVDMD with increasing inclusion, resulting in the strongest methane mitigation (57% reduction). Water hyacinth displayed a modestly low IVDMD and moderate methane reduction (14%). In conclusion, methane production is closely associated with substrate degradability, and each ingredient exhibits distinct functional tendencies: crushed almonds enhanced energy availability and digestibility, peanut skins showed methane mitigation potential, and water hyacinth functioned primarily as a low-fermentability grass hay substitute.

## Simple Summary

Livestock production systems are under pressure to deliver affordable feeds that sustain productivity while reducing greenhouse gas emissions. This study examined three unconventional feed and food by-products—crushed almonds, peanut skins, and water hyacinth—as feed additives and grass hay substitutes in ruminant diets. Almonds, with high energy and low fiber content, improved digestibility and fermentation but increased methane emissions, indicating both benefits and drawbacks. Peanut skins, a common agricultural by-product, reduced methane emissions by more than half, although digestibility declined at higher inclusion levels. Water hyacinth maintained digestibility similar to that of grass hay but showed comparatively limited fermentation activity. These findings suggest that these agricultural residues, especially peanut skins, could help pastoral farming systems and reduce methane emissions while partially replacing grass hay. Further animal trials and economic evaluations are required to confirm the practical implications; however, these resources hold promise for climate-smart animal agriculture.

## 1. Introduction

Ruminant livestock systems are under growing pressure to minimize their environmental impacts while sustaining productivity, particularly in areas with limited feed availability and inconsistent forage quality [1,2,3,4]. Enteric methane emissions have been extensively described as a major negative environmental factor, representing one of the most significant sources of greenhouse gases from ruminant livestock systems [5]. Methane is produced during anaerobic fermentation as the metabolic end product of hydrogen utilization by methanogenic archaea [6]. Extensive research has been directed toward dietary strategies that can modify rumen fermentation to reduce methane (CH_4_) output from ruminants, including lipid supplementation, plant secondary metabolites, and the use of alternative or effective low-cost feed resources [7,8].

Agro-industrial by-products, such as almond hulls, peanut skins, and tree nut residues, are promising feed ingredients owing to their availability, energy density, and bioactive compounds [9,10,11]. Likewise, invasive aquatic plants, such as *Eichhornia crassipes* (water hyacinth), offer potential as low-cost feed, particularly in tropical areas where they grow abundantly, while posing management challenges and raising environmental concerns [12]. Use of these materials supports circular economic goals by transforming waste into valuable ruminant feed, as highlighted in recent assessments of waste-derived feed substitutes [13]. However, their nutritional value and impact on fermentation vary widely due to differences in chemical compositions and processing. For instance, lipid-rich residues, such as almonds and peanuts, may reduce methanogenesis at high inclusion levels by decreasing protozoal populations, inhibiting hydrogen production pathways, and altering fiber digestion [14,15]. Furthermore, tannin-rich materials such as peanut skins may influence protein degradation, ammonia release, and fermentation patterns, thereby reducing microbial protein formation and methane output [16]. Water hyacinth, although fibrous and nutritionally limited, contains bioactive compounds such as phenolics, tannins, saponins, and flavonoids that may modulate rumen fermentation pathways, thereby reducing CH_4_ production through biochemical inhibition rather than solely through degradability [17]. Water hyacinth may serve as a low-cost roughage substitute at moderate inclusion levels; however, its practical application warrants caution, as aquatic plants are known to accumulate minerals and, in some environments, potentially harmful compounds such as heavy metals. Therefore, any in vivo use of water hyacinth should carefully consider the biomass source, mineral balance, and animal physiological responses. A crucial yet underexplored question is whether these materials exert different functional effects when used as feed additives (1, 2.5, 5, and 10%) or as replacement ingredients for conventional feed resources (25, 50, 75, and 100%).

Most previous studies have examined only one inclusion strategy, typically focusing on feed additives alone, thereby limiting comparative evaluation and constraining the development of integrated feeding and CH_4_-mitigation frameworks [18]. There is growing interest in using agro-residues and aquatic biomass as sustainable feed resources for ruminants worldwide. However, much of the existing research has focused on the chemical composition or in vitro digestibility of individual by-products, while fewer studies have conducted integrated comparative evaluations that simultaneously consider digestibility, rumen fermentation characteristics, and methane production. García-Rodríguez [19] and Goenaga [20] have begun to explore differences in fermentation kinetics and gas production among various by-products, underscoring the need for more systematic studies that explicitly incorporate methane dynamics. Notably, the functional impact of alternative feed resources depends not only on their chemical composition but also on their mode of dietary inclusion. When incorporated as feed additives, such materials primarily act as modifiers of rumen metabolism and may fail to substantially alter methane pathways unless inclusion thresholds are exceeded. In contrast, their use as structural replacements for conventional roughage fundamentally reshapes the rumen substrate environment by altering fiber availability, lipid supply, and protein degradability, thereby exerting more pronounced effects on fermentation dynamics and methanogenesis. Distinguishing between these two strategies is therefore critical for accurately evaluating both nutritional value and CH_4_ mitigation potential.

Therefore, the current study used two experimental designs to evaluate the chemical composition and nutritional properties of crushed almonds, peanut skins, and water hyacinth and to compare their effects on digestibility, rumen fermentation, and CH_4_ emission when incorporated at additive levels (1, 2.5, 5, and 10%) and grass hay replacement levels (25, 50, 75, and 100%). The choice of grass hay replacement in experiment 2 was intentional, as peanut skins and water hyacinth are structurally fibrous and more comparable to forage than to energy concentrates, such as crushed almonds. This design allowed us to assess their contribution to the roughage fraction, particularly the structural fiber composition and rumen fermentation dynamics. We therefore hypothesized that the use of these materials as additives would not alter digestibility or fermentation characteristics, but would reduce CH_4_ production, whereas their use as hay replacements would induce more pronounced, ingredient-specific responses due to differences in substrate composition (fiber content, lipid levels, and protein degradability), and that these ingredients could effectively replace grass hay and improve CH_4_ reduction potential.

## 2. Materials and Methods

The experiments were carried out at the Obihiro University of Agriculture and Veterinary Medicine, Japan, in accordance with the protocol approved by the Animal Care and Ethics Committee (approval No. 24–79). Donor cattle were housed, maintained, and managed at the University Field Science Center.

### 2.1. Composition of the Basal Diet and Experimental Samples

The basal rations consisted of grass hay, Kleingrass (*Panicum coloratum*) ground to a particle size of 1 mm (Retsch SM-2000, Retsch GmbH, Haan, North Rhine-Westphalia, Germany) and a commercial concentrate feed (Alpha Kotan, Chubu Feed Co., Ltd., Nagoya, Aichi, Japan). Three by-products were tested: crushed almonds, peanut skins (provided by Refine Holdings Co., Ltd., Gifu, Japan), and water hyacinth (*Eichhornia crassipes*; provided by Tropical Fish Shop Guppy, Obihiro, Hokkaido, Japan). Water hyacinth (whole plant: leaves, stems, and roots combined) was oven-dried at 60 °C for 48 h (DX 600, Yamato, Scientific, Tokyo, Japan) and milled with a Retsch SM-2000 cutting mill (Retsch GmbH, Haan, North Rhine-Westphalia, Germany) to pass through a 1 mm sieve. Peanut skins were obtained in a fine powdered form and used as received, while crushed almonds were ground to 1 mm using the same mill. The chemical compositions of the basal diet ingredients and experimental samples are presented in Table 1.

### 2.2. Donor Cows and Collection of Rumen Fluid

Rumen fluid was obtained from two non-lactating, approximately nine-year-old Holstein cows fitted with rumen fistulas, with an average body weight of 894 kg. The animals were housed with continuous access to fresh water and mineral lick blocks (KOENE250 TZ, Nippon Zenyaku Kogyo Co., Fukushima, Japan) and fed a maintenance-level diet of orchard grass (*Dactylis glomerata*). Approximately 1.3 L of rumen fluid was collected from four sites within the rumen of each cow. The fluid was strained through four layers of gauze, then pooled and transferred into a thermos flask preheated to 39 °C. Samples were delivered to the laboratory within 20 min, with the flask kept in an insulated container containing water maintained at 37–39 °C during transit.

### 2.3. Experimental Design and In Vitro Fermentation

Two separate in vitro experiments were conducted with different diets. In experiment 1, the control diet consisted of a basal ration with equal proportions of grass hay and concentrate (50:50), and each material (crushed almonds, peanut skins, and water hyacinth) was incorporated as a feed additive at different proportions (1, 2.5, 5, and 10%) of the substrate. In experiment 2, the control group was the same as that in experiment 1; however, each sample was introduced into the basal diet to replace grass hay at graded levels (25, 50, 75, and 100%). The replacement ratios used were 25:75 (12.5% sample + 37.5% grass hay + 50% concentrate), 50:50 (25% sample + 25% grass hay + 50% concentrate), 75:25 (37.5% sample + 12.5% grass hay + 50% concentrate), and 100 (50% sample + 50% concentrate). Each treatment in both experimental designs was incubated in triplicate, and the entire experiment was repeated in four independent runs conducted over different weeks. The in vitro batch culture procedure was performed according to the protocol established by Ahmed [21]. Briefly, approximately 500 mg of substrate was weighed into nylon bags (53 ± 10 μm pore size; BG1020, Sanshin Industrial Co., Ltd., Kanagawa, Japan), sealed, and placed in 120 mL fermentation bottles. In Experiment 1 (Exp. 1), feed additives were added directly to the bottles, whereas in Experiment 2 (Exp. 2), samples were placed inside nylon bags as part of the basal diet. Under continuous CO_2_ flushing, 40 mL of buffer solution (pH 6.8) was prepared according to McDougall [22] and mixed with 20 mL of rumen fluid in each fermentation bottle, including the blanks. Bottles were further flushed with CO_2_, sealed with butyl rubber stoppers and aluminum caps (Maruemu Co., Ltd., Osaka, Japan), and incubated at 39 °C for 24 h.

### 2.4. Fermentation Medium Sampling Procedures

After 24 h of incubation, the total gas volume was determined using calibrated VAN glass syringes (50 and 100 mL; TSUBASA Industry Co., Ltd., Tokyo, Japan). Headspace gas samples from each fermentation bottle were collected into vacutainer tubes (BD, Becton Drive, NJ, USA) and stored at room temperature until methane (CH_4_) and carbon dioxide (CO_2_) analysis. Following gas sampling, the bottle caps were removed, and the pH was immediately recorded using a LAQUA F-72 m (HORIBA Scientific, Kyoto, Japan). Approximately 1 mL of culture fluid was transferred into Eppendorf tubes (Eppendorf AG, Hamburg, Germany) and centrifuged at 16,000× *g* for 5 min at 4 °C. The resulting supernatant was retained and frozen at −20 °C for subsequent determination of volatile fatty acids (VFAs). Filter bags containing the residues were rinsed thoroughly with tap water until clear and then dried at 60 °C for 48 h to assess in vitro dry matter digestibility (IVDMD). Digestibility was calculated as the proportion of dry matter lost relative to the initial bag weight.

### 2.5. Chemical Characterization of the Samples

The chemical compositions of grass hay, concentrate mixture, and all experimental samples were analyzed at Obihiro University using the Association of Official Analytical Chemists (AOAC) standard methods [23]. Dry matter (DM) content was determined by oven-drying the samples at 135 °C for 2 h (method 930.15). Organic matter (OM) and ash were quantified by incineration in a muffle furnace at 500 °C for 3 h (method 942.05). Ether extract (EE) was measured following method 920.39. Nitrogen content was determined using the Kjeldahl procedure (method 984.13) with an electric digester and an automated distillation unit (DK 20, VELP Scientifica, Usmate (MB), Italy). Crude protein (CP) content was calculated as nitrogen × 6.25. Neutral detergent fiber (NDF), acid detergent fiber (ADF), and acid detergent lignin (ADL) were analyzed using amylase and sodium sulfite correction [24]. Non-fiber carbohydrates (NFC) were estimated using the following formula: NFC (%) = 100 − (NDF% + CP% + EE% + Ash%). Hemicellulose (HC) and cellulose (CEL) were calculated as HC% = NDF% − ADF% and CEL% = ADF% − ADL%, respectively. Total digestible nutrients (TDN), hemicellulose, and cellulose values for the basal diet and samples were derived using the National Research Council equations [25]. The chemical compositions of the experimental treatments in Experiment 2 are presented in Table 2.

### 2.6. Gas Profile Evaluation

The CH_4_ and CO_2_ concentrations were measured using a GC-8A gas chromatograph (Shimadzu Corp., Kyoto, Japan). A 1 mL aliquot of each sample was injected with a Hamilton gastight syringe (Hamilton Company, Reno, NV, USA), as per Ahmed [26].

### 2.7. Determination of VFAs

Volatile fatty acid (VFA) concentrations in the culture supernatants were quantified using high-performance liquid chromatography (LC-20; Shimadzu Corp., Kyoto, Japan) according to the protocol described by Ahmed [27].

### 2.8. Data Analysis

All datasets were processed using SAS software (version 9.4; SAS Institute Inc., Cary, NC, USA). A PROC MIXED model was applied, with treatment effects specified as fixed factors and experimental runs treated as random factors. The statistical model used was $Y_{ijk}=\mu +T_{i}+R_{j}+E_{ijk}$ where *Y_ijk_* represents the dependent variable, μ  is the overall mean, $T_{i}$ denotes the treatment effect, $R_{j}$ is the random effect of run, and $E_{ijk}$ is the residual error term. Results are expressed as mean values accompanied by pooled standard errors. Differences among the treatment means were evaluated using Tukey’s multiple comparison test. Statistical significance was declared at *p* < 0.05.

## 3. Results

The two in vitro trials revealed distinct responses depending on whether crushed almonds, peanut skins, and water hyacinth were used as feed additives (Exp. 1) or as grass hay replacements (Exp. 2). In Exp. 1, the addition of crushed almonds, peanut skins, and water hyacinth at levels of 1%, 2.5%, 5%, and 10% to the basal diet significantly increased total gas production, with the highest values in water hyacinth at 10% (48.92 mL) and crushed almonds at 5% (46.67 mL), both exceeding that of the control group (43.08 mL; *p =* 0.002; Table 3). The total gas per gram of digestible dry matter (mL/g DDM) also increased significantly across all inclusion levels, peaking at 10% water hyacinth (*p =* 0.006; Table 3). However, CH_4_ production ranged from 2.71 for peanut skins to 3.27 mL for water hyacinth at 10% inclusion and did not differ significantly compared with the control (2.89 mL; *p =* 0.279; Table 3). The tested ingredients at all levels of inclusion in the basal diet did not significantly alter the rumen fermentation profile and stayed within the physiological range; IVDMD varied from 41.81% for peanut skins at 10% to 44.49% for water hyacinth at 2.5% compared to the control of 43.13% (*p =* 0.999; Table 4). The pH was also not significant, with values between 6.66 and 6.72 compared to those of the control, 6.69 (*p =* 0.999; Table 4). Although CH_4_ per gram of DM and DDM increased with higher inclusion levels, particularly for crushed almonds and water hyacinth compared with the control group, there was no significant increase (*p =* 0.850 and *p =* 0.547, respectively; Table 4).

Exp. 2 showed that replacing grass hay with crushed almonds, peanut skins, and water hyacinth produced clear, ingredient and inclusion-dependent effects on digestibility, fermentation, and CH_4_ production (Table 5 and Table 6). Grass hay replacement significantly altered gas production and CH_4_ output (*p* < 0.001), but response patterns differed markedly among substrates. Crushed almonds consistently enhanced fermentative activity, characterized by increased total gas production and higher CH_4_ output at intermediate to high replacement levels. In contrast, peanut skins induced a progressive decline in gas production and CH_4_ emissions as the replacement level increased. Water hyacinth produced more moderate responses, with limited increases in gas production at partial replacement and reduced fermentability at complete substitution, when compared with the control. Digestibility responses diverged sharply among the test ingredients; crushed almond replacement significantly enhanced IVDMD, reaching the highest values at full replacement, whereas peanut skins and water hyacinth showed dose-dependent reductions in digestibility. These contrasting digestibility trends were accompanied by corresponding shifts in rumen fermentation characteristics and CH_4_ yield: crushed almonds at 50% replacement increased CH_4_ by 20% (2.91 mL), while complete replacement with peanut skins and water hyacinth reduced CH_4_ by 57% and 14%, respectively (*p <* 0.001; Table 5). These trends indicated that grass hay replacement fundamentally reshaped rumen fermentation dynamics in an ingredient-specific manner.

The lowest digestibility was observed at 100% replacement (peanut skins: 26.53%; water hyacinth: 42.32%; Table 6). Total VFA concentrations declined significantly across all treatments, with the lowest observed in 100% peanut skins compared to the control (*p <* 0.006; Table 5). Interestingly, both acetate and propionate (mol/100 mol) levels were significantly altered in all dietary treatments (*p =* 0.001 and *p <* 0.001, respectively; Table 6). Conversely, crushed almonds reduced the acetate-to-propionate ratio at all inclusion levels, while peanut skins significantly increased this ratio, with the highest value observed at 100% inclusion. In contrast, water hyacinth maintained an acetate-to-propionate ratio comparable to that of the control diet (*p <* 0.001; Table 6).

## 4. Discussion

Crushed almonds contained an exceptionally high ether extract (38.38%) and organic matter (97.51%) content. This is consistent with earlier reports that almond by-products contained substantial lipid and soluble carbohydrate fractions [28]. The crude protein content (22.47%) recorded in the current study, which typically ranged from 5.4 to 6.7% of dry matter, was higher than the values previously reported for almond by-products. This difference likely reflects varietal effects, processing factors, or the inclusion of kernel residues in the test material [29]. The relatively low fiber fractions (NDF 10.22%, ADF 7.22%), compared to the other test ingredients, suggest higher potential digestibility and fermentability. Thus, this compositional profile is consistent with the high TDN (83.03%) and aligns with the greater fermentability observed during in vitro incubation, as feed with lower structural fibers generally improved energy availability [30]. Additionally, total VFA concentrations in the crushed almond diets remained comparable to those of the control across all inclusion levels, indicating sustained ruminal fermentation activity and efficient utilization of digestible substrates, which is typical of feeds rich in readily fermentable carbohydrates and energy-dense components [29,31]. Peanut skins presented a moderate nutrient profile, with crude protein (13.87%) and ether extract (29.6%) values comparable to those reported in a recent meta-analysis of peanut by-products [32]. Its relatively high NDF (31.47%) and lignin content (4.24%) reflect the fibrous nature of peanut skins [33]. Water hyacinth showed the lowest crude protein content (7.27%) among all test ingredients, which is consistent with the lower range reported for whole-plant samples in previous studies [34,35] but lower than the leaf-only fractions [36,37]. Its high NDF (47.26%), ADF (19.86%), and hemicellulose (27.4%) contents indicate a substantial structural carbohydrate fraction, which is typical of water hyacinth harvested at full maturity, as reported for aerial biomass of this aquatic macrophyte [38,39]. The relatively high ash content (11.61%) reflects the capacity of aquatic plants to accumulate minerals from their growing environment, a characteristic feature consistently reported for water hyacinth and other floating macrophytes [39,40]. This combination of elevated mineral content and a complex fiber matrix may partially limit microbial accessibility, contributing to moderated fermentation responses [41,42]. Despite its low protein content, the TDN value (73.21%) suggested moderate fermentability, likely due to its soluble carbohydrate fraction (NFC 32.48%). Hossain [35] reported similar results, highlighting the high mineral content and limited nutritional value of water hyacinth. Celeste [43] demonstrated that ensiling water hyacinth improved its nutrient profile but still restricted its use compared to traditional forages.

The inclusion of agro-industrial by-products and aquatic biomass as feed additives did not negatively affect rumen digestibility or fermentation processes. This is consistent with earlier reports indicating that moderate amounts of lipid- or fiber-rich additives can sustain microbial activity without interfering with fermentation [44,45]. The slight increase in gas and CH_4_ production at higher inclusion rates might be due to increased availability of fermentable substrates, especially from crushed almonds, which are high in NFC and EE. Despite the increase in gas production, there was no significant reduction in CH_4_ emissions, indicating that CH_4_ output was unaffected by the feed additive inclusion levels of all the samples. This implies that the additives did not measurably alter the suppression of methanogenic archaea or redirect hydrogen to other pathways, such as propionate. The proportions of acetate, propionate, and butyrate remained unchanged, leading to no significant changes in the acetate-to-propionate (A:P) ratio. The stable A:P ratio and CH_4_/CO_2_ values support this interpretation, suggesting that hydrogen partitioning patterns remained unchanged, a conclusion consistent with several in vitro additive studies [46,47,48]. Overall, the additives did not adversely affect ruminal fermentation at any of the levels tested.

The substitution of grass hay with agricultural residues showed varied effects on rumen fermentation, highlighting the differences in their chemical composition and secondary metabolites. Crushed almonds consistently improved digestibility and gas production owing to their high TDN (83.2%) and low fiber content (NDF of 10.22%), which promotes rapid microbial fermentation and increased substrate availability [49]. Digestibility increased steadily, reaching approximately 64.7% at full replacement, consistent with findings that almond by-products enhance rumen fermentation through fermentable carbohydrates [50,51]. Although crushed almonds contain appreciable ether extract, which is often associated with methane suppression, the elevated supply of fermentable substrates and improved digestibility likely intensified overall microbial activity and hydrogen release, thereby overriding lipid-mediated inhibitory effects on methanogenesis. This distinction between lipid-associated inhibition and substrate-driven hydrogen production explains the observed increase in CH_4_ emissions (12%) at higher replacement levels. Similar findings have been reported where carbohydrate-rich by-products stimulated fermentation sufficiently to offset potential lipid effects on CH_4_ reduction [28,52,53]. Consequently, while crushed almonds represent a highly digestible, energy-rich feed resource, their use at high replacement levels requires careful dietary balancing to prevent unintended increases in CH_4_ production. Peanut skins showed a dose-dependent decline in IVDMD, likely due to their elevated ADL (4.24%) and ADF (19.05%) contents, which limited microbial access to structural carbohydrates. At higher replacement levels, digestibility decreased sharply, a pattern consistent with the presence of polyphenolic compounds in peanut by-products that are known to bind dietary protein and reduce microbial degradation efficiency. Water hyacinths exhibited a different pattern. IVDMD decreased from 46.26% at 25% replacement to 42.32% at full replacement, indicating only a smaller reduction in fermentability compared with the sharper decline observed for peanut skins. Despite its moderate fiber content, the high ash content (11.61%) and complex fiber fractions of water hyacinth were associated with reduced fermentability and lower digestibility [34,43]. Total VFA production was lower with water hyacinth replacement, although the pattern was not strictly dose-dependent, whereas A:P ratios shifted upward, suggesting reduced glycolytic flux toward propionate, a common outcome of low-quality roughages [54]. Gas production closely tracked digestibility for crushed almonds and peanut skins, whereas water hyacinth showed a relatively higher gas output per unit of digestibility, indicating less efficient fermentation of the substrate. CH_4_ production showed strong, significant treatment differences. Peanut skins progressively reduced CH_4_ emissions, with reductions up to 57% observed at full replacement. Similar CH_4_-lowering effects have been reported in goats and cattle consuming tannin-rich peanut residues [33,55]. However, as condensed tannins, phenolic compounds, protozoal populations, and hydrogen balance were not directly measured in this present study, the observed CH_4_ suppression should be interpreted as associative rather than mechanistic. The reduction in CH_4_ production is therefore from the compositional characteristics of peanut skins and the observed fermentation responses, in line with previous linking of tannin- and lignin-rich feeds with altered rumen fermentation pathways. [33,47,55,56,57,58]. Water hyacinth led to a slight reduction in CH_4_ (up to 14%), largely due to nutrient dilution and lower fermentability rather than active methanogenic properties. This pattern aligns with the findings of Celeste [43], Sondakh [59], and other in vivo studies [34,60], who attributed its limited CH_4_ mitigating effect to low fermentability. Overall, the findings of the current study are aligned with earlier studies: crushed almonds are digestible and energy-rich but increase methane levels; peanut skins, rich in tannins and lignin, effectively suppress CH_4_ but at the cost of digestibility; and water hyacinth, despite being bulky and mineral-rich, reduces fermentation efficiency with only a slight decrease in CH_4_ production. Methane reduction observed at high replacement levels indicates the potential for climate-smart feeding strategies, though the balance between digestibility and VFA production needs consideration. Combining these residues with more fermentable forages or adding microbial enhancers can enhance both energy output and environmental benefits [14,61].

The contrasting responses observed between the additive (Exp. 1) and feed replacement (Exp. 2) treatments highlight the fundamental differences in how substrates interact with the rumen environment. When included as additives, the test ingredients contributed insufficient amounts of lipids or secondary compounds to substantially modify the fermentation pathways, corresponding with previous observations that lipid-mediated CH_4_ inhibition generally requires higher inclusion rates [7]. In contrast, grass hay replacement altered the overall dietary nutrient matrix, resulting in distinct, ingredient-specific effects on rumen fermentation and CH_4_ production. Crushed almonds enhanced fermentability but increased CH_4_ production, whereas peanut skins suppressed fermentability and achieved the greatest CH_4_ mitigation, and water hyacinth produced moderate reductions driven by limited degradability. Collectively, these findings indicate that substrate substitution exerts a far stronger influence on rumen fermentation dynamics than does low-dose additive supplementation.

## 5. Conclusions

This study is, to the best of our knowledge, the first to demonstrate inclusion-dependent differences in ruminal fermentation responses to crushed almonds, peanut skins, and water hyacinth when used as feed additives or grass hay replacements. This study shows that dietary inclusion strategy, rather than ingredient identity alone, governs rumen fermentation and CH_4_ responses. When used as additives, they did not distinctively affect CH_4_ production, whereas grass hay replacement fundamentally altered fermentation dynamics, resulting in ingredient-specific outcomes. Crushed almonds improved digestibility but increased CH_4_ by 12%. Conversely, peanut skins reduced CH_4_ in a dose-dependent manner, up to a 57% reduction at full replacement. Water hyacinth yielded only modest CH_4_ reduction (up to 14%). Based on the observed responses, partial replacement levels (≤25–50%) appear more realistic for practical feeding, providing digestibility enhancement (crushed almonds) or CH_4_ mitigation (peanut skins) without severe fermentation penalties. Water hyacinth may serve as a low-cost roughage substitute at moderate inclusion levels. Agro-residues and aquatic biomass can support climate-smart feeding strategies, with each ingredient offering distinct advantages. Further in vivo validation and economic assessment are required to confirm practical applicability and feeding safety.

## Figures and Tables

**Table 1:** The chemical composition (%) of the basal diet ingredients and the experimental samples.

	Klein Grass Hay	Concentrate Mixture	Crushed Almonds	Peanut Skins	Water Hyacinth
Dry matter	89.73	86.75	95.71	92.61	89.58
Organic matter	90.42	95.93	97.51	97.89	88.39
Crude ash	9.58	4.07	3.30	2.11	11.61
Crude protein	11.00	17.81	22.47	13.87	7.27
Ether Extract	1.73	2.33	38.38	29.60	1.38
Neutral detergent fiber	63.35	24.28	10.22	31.47	47.26
Acid detergent fiber	34.28	7.43	7.22	19.05	19.86
Acid detergent lignin	4.47	1.11	1.95	4.24	2.04
Non-fiber carbohydrate	14.34	51.51	25.63	22.95	32.48
Hemicellulose	29.07	16.85	3.00	12.42	27.40
Cellulose	29.81	6.32	5.27	14.81	17.82
Total digestible nutrient	61.82	83.03	83.20	73.85	73.21

**Table 2:** Proximate chemical composition (% in dry matter) of the experimental treatments with inclusion levels of test ingredients used as grass hay replacement in the basal diet (Trial 2).

	Control	Crushed Almonds				Peanut Skins				Water Hyacinth			
Parameter	0%	25%	50%	75%	100%	25%	50%	75%	100%	25%	50%	75%	100%
Dry matter	88.24	88.99	89.74	90.48	91.23	88.60	88.96	89.32	89.68	88.22	88.20	88.18	88.17
Organic matter	93.18	94.06	94.95	95.83	96.72	94.11	95.04	95.98	96.91	92.92	92.67	92.41	92.16
Crude ash	6.83	6.04	5.26	4.47	3.69	5.89	4.96	4.02	3.09	7.08	7.33	7.59	7.84
Crude protein	14.41	15.84	17.27	18.71	20.14	14.76	15.12	15.48	15.84	13.94	13.47	13.01	12.54
Ether Extract	2.03	6.61	11.19	15.77	20.36	5.51	9.00	12.48	15.97	1.99	1.94	1.90	1.86
Neutral detergent fiber	43.82	37.17	30.53	23.89	17.25	39.83	35.85	31.86	27.88	41.80	39.79	37.78	35.77
Acid detergent fiber	20.86	17.47	14.09	10.71	7.33	18.95	17.05	15.14	13.24	19.05	17.25	15.45	13.65
Acid detergent lignin	2.79	2.48	2.16	1.85	1.53	2.76	2.73	2.70	2.68	2.49	2.18	1.88	1.58
Non-fiber carbohydrate	32.93	34.34	35.75	37.16	38.57	34.00	35.08	36.15	37.23	35.19	37.46	39.73	42.00
Hemicellulose	22.96	19.70	16.44	13.18	9.93	20.88	18.80	16.72	14.64	22.75	22.54	22.33	22.13
Cellulose	18.07	15.00	11.93	8.86	5.80	16.19	14.32	12.44	10.57	16.57	15.07	13.57	12.07
Total digestible nutrient	72.43	75.10	77.77	80.44	83.12	73.93	75.43	76.94	78.44	73.85	75.27	76.70	78.12

**Table 3:** Gas production responses to feed additive inclusion levels of the samples (1–10%) from 24 h in vitro incubation (Trial 1) (*n* = 12).

	Control	Crushed Almonds				Peanut Skins				Water Hyacinth					
Parameter	0%	1%	2.5%	5%	10%	1%	2.5%	5%	10%	1%	2.5%	5%	10%	SEM	*p* Value
Total gas (mL/g)	43.08 ^b^	43.92 ^b^	44.67 ^ab^	46.67 ^ab^	45.08 ^ab^	43.67 ^b^	43.25 ^b^	44.92 ^ab^	43.42 ^b^	43.83 ^b^	47.33 ^ab^	45.08 ^ab^	48.92 ^a^	0.95	0.002
Total gas/DM ^1^ (mL/g)	194.27	200.18	200.08	207.3	204.04	196.9	196.24	204.02	201.96	197.12	209.33	205.10	216.94	7.40	0.738
Total gas/DDM ^2^ (mL/g)	94.31 ^b^	96.47 ^ab^	98.38 ^ab^	102.91 ^ab^	99.20 ^ab^	95.92 ^ab^	95.04 ^b^	98.50 ^ab^	95.22 ^b^	96.68 ^ab^	103.88 ^ab^	99.03 ^ab^	107.38 ^a^	2.49	0.006
CH_4_ (%)	6.78	6.35	6.82	6.88	6.54	6.59	6.25	6.40	6.47	6.57	6.70	7.34	6.66	0.38	0.890
CO_2_ (%)	93.22	93.65	93.18	93.12	93.46	93.41	93.75	93.6	93.53	93.43	93.30	92.66	93.34	0.38	0.890
CH_4_ (mL/g)	2.89	2.78	3.06	3.22	2.95	2.89	2.71	2.88	2.82	2.88	3.17	3.26	3.27	0.17	0.279
CO_2_ (mL/g)	40.19 ^b^	41.13 ^b^	41.61 ^ab^	43.45 ^ab^	42.13 ^ab^	40.78 ^b^	40.54 ^b^	42.03 ^ab^	40.60 ^b^	40.95 ^b^	44.16 ^ab^	41.83 ^ab^	45.65 ^a^	0.91	0.007
CH_4_/CO_2_ (mL/g)	0.07	0.07	0.07	0.07	0.07	0.07	0.07	0.07	0.07	0.07	0.07	0.08	0.07	0.00	0.866
CH_4_/DM (mL/g)	13.14	12.74	13.76	14.39	13.43	13.12	12.35	13.24	13.23	13.03	14.15	15.15	14.63	1.05	0.850
CH_4_/DDM (mL/g)	6.37	6.17	6.80	7.16	6.54	6.40	5.98	6.37	6.23	6.38	7.00	7.22	7.21	0.45	0.547
CO_2_/DM (mL/g)	181.13	187.44	186.32	192.91	190.61	183.78	183.89	190.78	188.73	184.10	195.18	189.95	202.31	6.69	0.731
CO_2_/DDM (mL/g)	87.94 ^b^	90.30 ^ab^	91.58 ^ab^	95.76 ^ab^	92.65 ^ab^	89.52 ^b^	89.06 ^b^	92.13 ^ab^	88.99 ^b^	90.30 ^ab^	96.88 ^ab^	91.81 ^ab^	100.17 ^a^	2.20	0.004

^1^ DM: Dry matter. ^2^ DDM: Digestible dry matter. SEM: Standard error of the mean. ^a,b^ Values with different superscripts in the same row are significantly different (*p* < 0.05).

**Table 4:** Rumen fermentation responses to feed additive inclusion levels of the samples (1–10%) from 24 h in vitro incubation (Trial 1) (*n* = 12).

	Control	Crushed Almonds				Peanut Skins				Water Hyacinth					
Parameter	0%	1%	2.5%	5%	10%	1%	2.5%	5%	10%	1%	2.5%	5%	10%	SEM	*p* Value
pH	6.69	6.70	6.72	6.69	6.68	6.67	6.67	6.69	6.69	6.68	6.67	6.66	6.66	0.05	0.999
IVDMD ^1^ (%)	43.13	42.95	44.18	44.42	43.31	43.48	42.95	43.01	41.81	43.77	44.49	42.84	43.71	1.70	0.999
Acetate (mmol/L)	40.94	40.43	41.22	42.25	41.44	39.34	40.34	41.33	40.77	39.31	39.70	40.05	42.11	1.68	0.983
Propionate (mmol/L)	15.26	15.40	15.49	16.12	15.98	14.55	15.09	15.56	15.12	14.79	14.94	15.08	15.80	0.46	0.453
Butyrate (mmol/L)	6.28	6.16	6.40	6.60	6.32	6.02	6.17	6.34	6.21	6.02	6.20	6.14	6.43	0.24	0.931
TVFA ^2^ (mmol/L)	62.48	61.99	63.12	64.96	63.73	59.92	61.61	63.24	62.09	60.11	60.84	61.27	64.34	2.18	0.904
Acetate (mol/100 mol)	65.47	65.18	65.20	64.93	64.90	65.47	65.36	65.25	65.50	65.29	65.14	65.24	65.36	0.66	1.000
Propionate (mol/100 mol)	24.49	24.91	24.65	24.91	25.18	24.49	24.62	24.72	24.51	24.73	24.69	24.74	24.67	0.61	0.999
Butyrate (mol/100 mol)	10.04	9.92	10.14	10.16	9.92	10.05	10.02	10.03	9.99	9.98	10.17	10.02	9.96	0.12	0.934
A/P ^3^ ratio (mmol/L)	2.70	2.64	2.67	2.63	2.60	2.70	2.68	2.66	2.70	2.66	2.66	2.66	2.68	0.09	1.000

^1^ IVDMD: In vitro dry matter digestibility. ^2^ TVFA: Total volatile fatty acid. ^3^ A/P: Acetate/propionate. SEM: Standard error of the mean.

**Table 5:** Gas production responses to grass hay replacement of different inclusion levels of the samples (25–100%) from the 24 h in vitro incubation (*n* = 12) (Trial 2).

	Control	Crushed Almonds				Peanut Skins				Water Hyacinth					
Parameter	0%	25%	50%	75%	100%	25%	50%	75%	100%	25%	50%	75%	100%	SEM	*p* Value
Total gas (mL/g)	40.50 ^abc^	42.50 ^ab^	43.63 ^a^	40.54 ^abc^	40.75 ^abc^	37.38 ^bc^	34.00 ^cd^	28.96 ^de^	23.63 ^e^	42.38 ^ab^	42.50 ^ab^	41.00 ^abc^	39.58 ^abc^	1.27	<0.001
Total gas/DM ^1^ (mL/g)	171.31 ^a–d^	169.15 ^a–d^	158.81 ^b–e^	150.24 ^de^	135.54 ^e^	182.11 ^ab^	181.28 ^abc^	168.15 ^a–d^	153.72 ^cde^	183.00 ^ab^	188.07 ^a^	183.73 ^ab^	182.24 ^ab^	5.91	<0.001
Total gas/DDM ^2^ (mL/g)	91.97 ^ab^	97.53 ^ab^	99.00 ^a^	91.77 ^ab^	93.02 ^ab^	84.84 ^bc^	77.04 ^cd^	65.58 ^de^	53.62 ^e^	96.19 ^ab^	96.37 ^ab^	92.78 ^ab^	89.39 ^abc^	2.87	<0.001
CH_4_ (%)	6.03 ^abc^	6.41 ^ab^	6.68 ^a^	6.09 ^abc^	6.72 ^a^	5.26 ^c–f^	5.01 ^def^	4.61 ^f^	4.43 ^f^	5.98 ^abc^	5.77 ^bcd^	5.52 ^b–e^	5.29 ^c–f^	0.19	<0.001
CO_2_ (%)	93.97 ^def^	93.59 ^ef^	93.32 ^f^	93.91 ^def^	93.28 ^f^	94.74 ^a–d^	94.99 ^abc^	95.39 ^ab^	95.57 ^a^	94.02 ^def^	94.23 ^cde^	94.48 ^b–e^	94.71 ^a–d^	0.19	<0.001
CH_4_ (mL/g)	2.43 ^bcd^	2.73 ^ab^	2.91 ^a^	2.48 ^bcd^	2.73 ^ab^	1.95 ^ef^	1.69 ^fg^	1.33 ^gh^	1.04 ^h^	2.52 ^abc^	2.44 ^bcd^	2.26 ^cde^	2.09 ^def^	0.09	<0.001
CO_2_ (mL/g)	38.07 ^a^	39.77 ^a^	40.71 ^a^	38.07 ^a^	38.02 ^ab^	35.42 ^ab^	32.31 ^bc^	27.63 ^cd^	22.58 ^d^	39.85 ^a^	40.06 ^a^	38.74 ^a^	37.50 ^ab^	1.22	<0.001
CH_4_/CO_2_ (mL/g)	0.06 ^abc^	0.07 ^ab^	0.07 ^a^	0.07 ^abc^	0.07 ^a^	0.06 ^c–f^	0.05 ^def^	0.05 ^ef^	0.05 ^f^	0.06 ^abc^	0.06 ^bcd^	0.06 ^cde^	0.06 ^c–f^	0.00	<0.001
CH_4_/DM (mL/g)	10.26 ^a^	10.81 ^a^	10.59 ^a^	9.20 ^ab^	9.12 ^ab^	9.52 ^ab^	9.06 ^ab^	7.74 ^bc^	6.80 ^c^	10.90 ^a^	10.81 ^a^	10.10 ^a^	9.61 ^ab^	0.40	<0.001
CH_4_/DDM (mL/g)	5.51 ^bcd^	6.25 ^ab^	6.61 ^a^	5.61 ^bcd^	6.22 ^ab^	4.43 ^ef^	3.84 ^fg^	3.02 ^gh^	2.37 ^h^	5.72 ^abc^	5.54 ^bcd^	5.11 ^cde^	4.71 ^def^	0.21	<0.001
CO_2_/DM (mL/g)	161.05 ^abc^	158.35 ^abc^	148.23 ^bcd^	141.03 ^cd^	126.42 ^d^	172.59 ^ab^	172.22 ^ab^	160.42 ^abc^	146.92 ^bcd^	172.10 ^ab^	177.26 ^a^	173.63 ^ab^	172.62 ^ab^	5.67	<0.001
CO_2_/DDM (mL/g)	86.46 ^a^	91.28 ^a^	92.40 ^a^	86.17 ^ab^	86.80 ^a^	80.40 ^ab^	73.20 ^bc^	62.56 ^cd^	51.26 ^d^	90.46 ^a^	90.83 ^a^	87.67 ^a^	84.68 ^ab^	2.76	<0.001

^1^ DM: Dry matter. ^2^ DDM: Digestible dry matter. SEM: Standard error of the mean. ^a–h^ Values with different superscripts in the same row are significantly different (*p* < 0.05).

**Table 6:** Rumen fermentation responses to grass hay replacement of different inclusion levels of the samples (25–100%) from the 24 h in vitro incubation (*n* = 12) (Trial 2).

	Control	Crushed Almonds				Peanut Skins				Water Hyacinth					
Parameter	0%	25%	50%	75%	100%	25%	50%	75%	100%	25%	50%	75%	100%	SEM	*p* Value
pH	6.63 ^ab^	6.63 ^ab^	6.62 ^ab^	6.63 ^ab^	6.63 ^ab^	6.61 ^abc^	6.63 ^ab^	6.66 ^a^	6.72 ^a^	6.59 ^abc^	6.51 ^bc^	6.48 ^c^	6.51 ^bc^	0.03	<0.001
IVDMD ^1^ (%)	47.47 ^de^	52.06 ^cd^	57.25 ^b^	56.11 ^bc^	64.74 ^a^	39.51 ^g^	35.18 ^gh^	31.28 ^hi^	26.53 ^i^	46.26 ^ef^	44.83 ^ef^	43.94 ^efg^	42.32 ^fg^	1.07	<0.001
Acetate (mmol/L)	40.34	39.59	39.73	37.67	38.29	35.30	35.88	32.98	31.95	38.20	34.51	40.33	37.45	1.97	0.030
Propionate (mmol/L)	14.82 ^a^	14.69 ^a^	14.91 ^a^	14.22 ^a^	14.86 ^a^	12.54 ^abc^	12.99 ^ab^	10.55 ^bc^	9.67 ^c^	14.24 ^a^	12.54 ^abc^	14.21 ^a^	13.63 ^a^	0.65	<0.001
Butyrate (mmol/L)	6.06 ^a^	6.10 ^a^	6.27 ^a^	5.90 ^ab^	6.20 ^a^	5.12 ^ab^	5.10 ^ab^	4.53 ^b^	5.02 ^ab^	5.78 ^ab^	5.25 ^ab^	5.82 ^ab^	5.59 ^ab^	0.32	0.001
TVFA ^2^ (mmol/L)	61.22 ^a^	60.38 ^ab^	60.92 ^ab^	57.80 ^abc^	59.35 ^abc^	52.96 ^abc^	53.96 ^abc^	48.05 ^bc^	46.64 ^c^	58.22 ^abc^	52.30 ^abc^	60.35 ^ab^	56.67 ^abc^	2.77	0.006
Acetate (mol/100 mol)	65.86 ^abc^	65.48 ^bc^	65.17 ^c^	65.03 ^c^	64.41 ^c^	66.51 ^abc^	66.65 ^abc^	68.42 ^a^	68.23 ^ab^	65.42 ^bc^	65.89 ^abc^	66.47 ^abc^	65.97 ^abc^	0.62	0.001
Propionate (mol/100 mol)	24.22 ^ab^	24.38 ^a^	24.51 ^a^	24.72 ^a^	25.10 ^a^	23.79 ^ab^	23.88 ^ab^	22.10 ^bc^	20.79 ^c^	24.62 ^a^	24.20 ^ab^	23.82 ^ab^	24.15 ^ab^	0.48	<0.001
Butyrate (mol/100 mol)	9.91	10.13	10.32	10.25	10.50	9.70	9.47	9.47	10.98	9.96	9.91	9.71	9.88	0.51	0.759
A/P ^3^ ratio	2.73 ^c^	2.70 ^c^	2.67 ^c^	2.64 ^c^	2.57 ^c^	2.81 ^bc^	2.83 ^bc^	3.12 ^ab^	3.29 ^a^	2.67 ^c^	2.73 ^c^	2.83 ^bc^	2.74 ^c^	0.07	<0.001

^1^ IVDMD: In vitro dry matter digestibility. ^2^ TVFA: Total volatile fatty acid. ^3^ A/P: Acetate/propionate. SEM: Standard error of the mean. ^a–i^ Values with different superscripts in the same row are significantly different (*p* < 0.01).

## Data Availability

Supporting data for the findings reported in this study can be obtained upon request from the corresponding authors.
